# Giant gastrointestinal stromal tumor (GIST) of the stomach cause of high bowel obstruction: surgical management

**DOI:** 10.1186/1477-7819-11-172

**Published:** 2013-08-05

**Authors:** Alessandro Cappellani, Gaetano Piccolo, Francesco Cardì, Andrea Cavallaro, Emanuele Lo Menzo, Vincenzo Cavallaro, Antonio Zanghì, Maria Di Vita, Massimiliano Berretta

**Affiliations:** 1 Department of Surgery, University of Catania, Via S. Sofia 78, Catania 95123, Italy; 2 Digestive Disease Institute, Cleveland Clinic Florida, Weston, FL, USA; 3 Department of Surgical Sciences, Transplantation and Advanced Technologies, University of Catania, Via S. Sofia n.84, Catania 95123, Italy; 4 Department of Medical Oncology, National Cancer Institute, Aviano, PN, Italy

**Keywords:** Giant, GIST, Surgical management

## Abstract

**Background:**

Gastrointestinal stromal tumors (GISTs) represent 85% of all mesenchymal neoplasms that affect the gastrointestinal (GI) tract. These GISTs range in size from small lesions to large masses. Often they are clinically silent until they reach a significant size, so their discovery is usually incidental.

**Case presentation:**

A 67-year-old man was admitted at our general surgery department with a persistent abdominal pain in the left hypochondrium, associated with nausea and vomiting. Clinical examination revealed a palpable mass in the epigastrium and in the left hypochondrium, which was approximately 40 cm long. Ultrasonography and computed tomography of the abdomen showed a large mass of 40 × 25 cm, which extended from the posterior wall of the stomach to the spleen, involving the body and the tail of the pancreas. The patient underwent en-block resection of the mass, sleeve resection of the stomach, and distal pancreatectomy-splenectomy. The histopathology of the resected specimen was consistent with a gastrointestinal stromal tumor of the stomach (positive for CD 117) with a high risk of malignancy (mitotic count >5/50 high-power fieldand Ki67/Mib1 >10%). The postoperative course was uneventful and treatment with imatinib mesylate began immediately. The patient appears to be disease free after four years.

**Conclusions:**

Giant GISTs of the stomach are rare. Surgical resection with curative intent is feasible. The combination of surgical resection and imatinib can provide long-termdisease-free survival. An R0 resection is the best achievable treatment, therefore the patient should be evaluated over time for potential resectability.

## Background

Gastrointestinal stromal tumors (GISTs) represent 85% of all mesenchymal neoplasms that affect the gastrointestinal (GI) tract [1].

In the past, these tumors were classified as leiomyomas, leiomyosarcomas, or leiomyoblastomas. Only recently, with the help of immunohistochemistry, have GISTs become considered a separate entity. These tumors are believed to arise from the KIT (CD117) positive interstitial cell of Cajal, the pacemaker cell of the GI tract [2,3].

Approximately 85% of GISTs harbor an activating *KIT* mutation, which leads to constitutive activation of *KIT* and its tyrosine kinase function. Approximately 3% to 5% of GISTs instead carry a mutation in the *PDGFRa* gene, and about 10% to 15% of the tumors contain wild-type forms of the *KIT* and *PDGFRa* proto-oncogenes [4-6].

These tumors are located primarily in the stomach (60% to 70%) and their discovery is often incidental [7]. GISTs range in size from small lesions to large masses. They are clinically silent until they reach a significant size; this is why their discovery is usually incidental [1]. In many cases, GISTs present with abdominal pain, GI bleeding or palpable mass. In other cases, they may be revealed because of a complication: bowel obstruction, spontaneous rupture into the peritoneal cavity leading to peritonitis, or tumor rupture into the stomach [8-10]. We report an unusual case of a giant GIST that caused a proximal bowel obstruction.

## Case presentation

A 67-year-old man was admitted to the Department of General Surgery of the University of Catania with a persistent abdominal pain in the left hypochondrium associated with nausea, vomiting, and weight loss (5 kg in two months). Clinical examination revealed a palpable mass in the epigastrium and in the left hypochondrium, which was approximately 40 cm long. The abdominal mass appeared to be fixed to adjacent structures, with its limits poorly defined.

**Figure 1:**
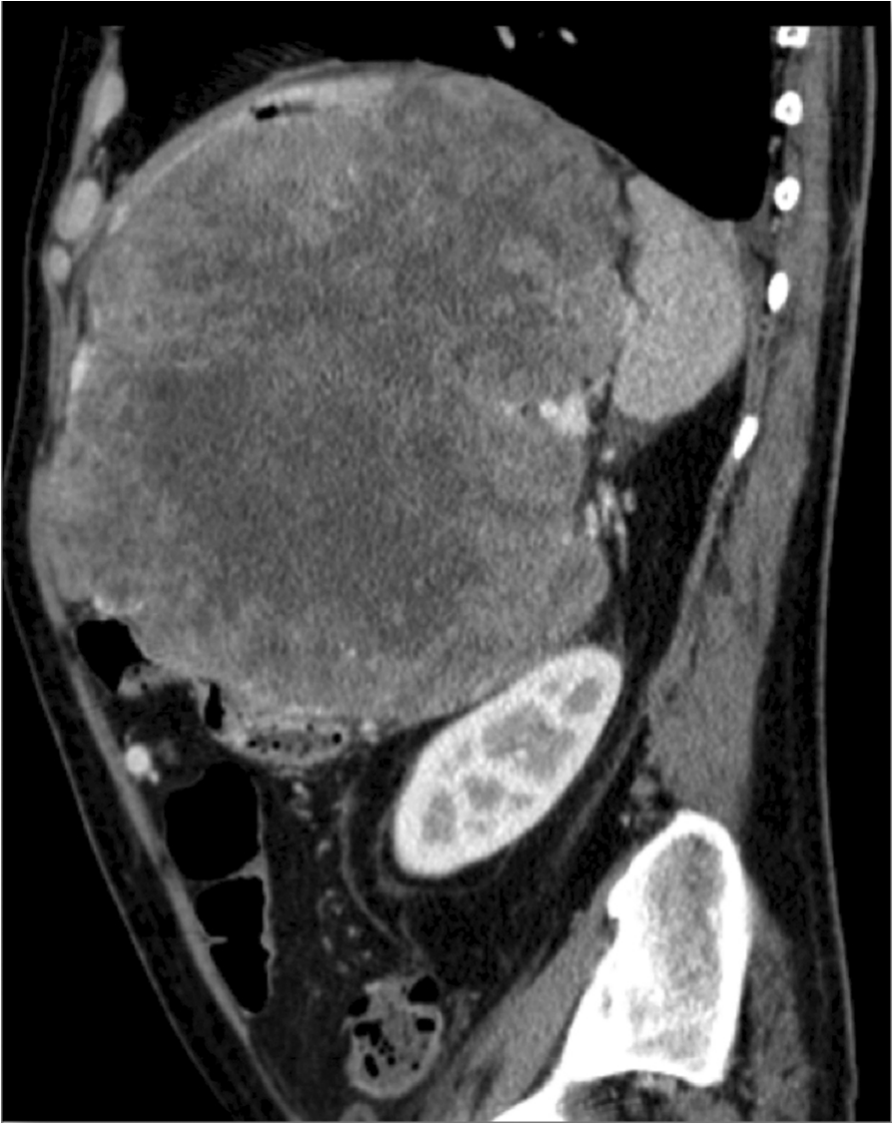
Sagittal computed tomogram, showing the extent of tumor and the compression of the left colonic flexure.

**Figure 2:**
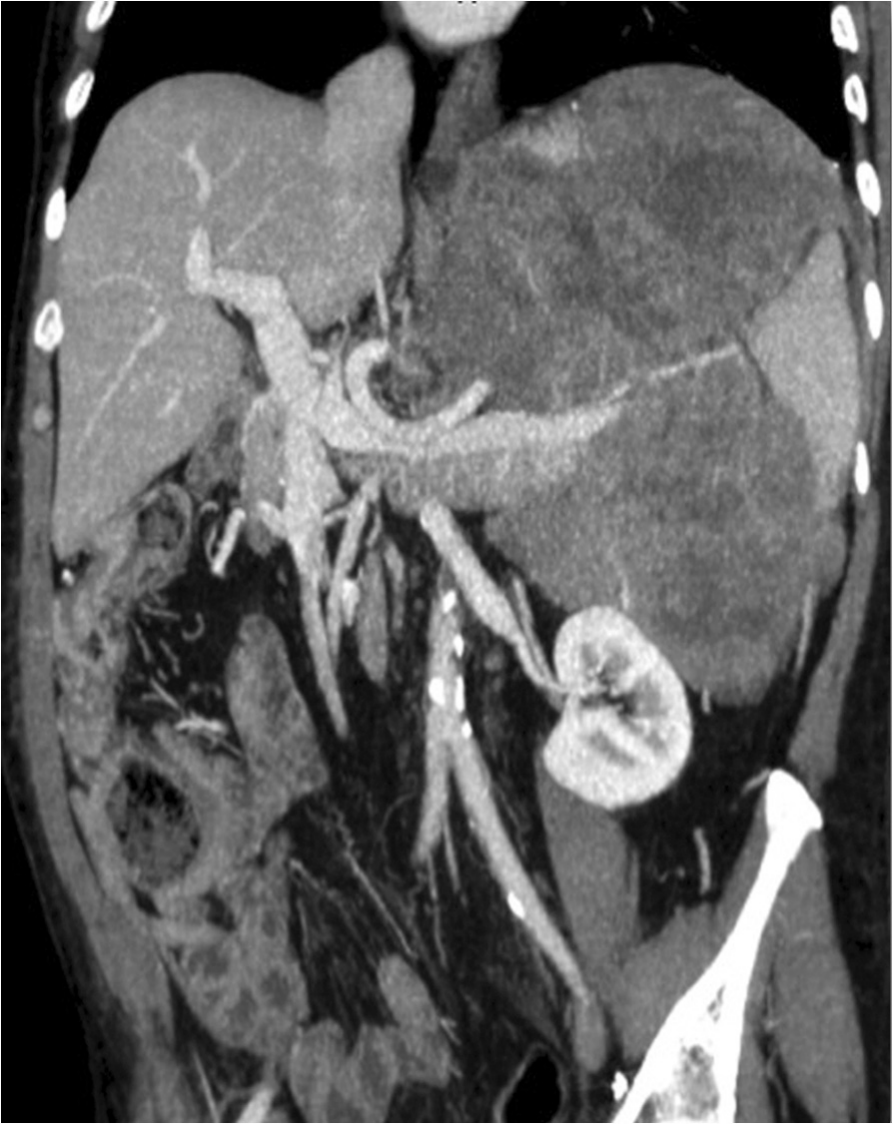
Coronal computed tomogram, showing a large mass of 40 × 25 cm, which extended from the posterior wall of the stomach to the spleen, enveloping the body and the tail of the pancreas.

All routine blood test results and levels of tumor markers (CEA, CA 19–9, α-fetoprotein) were within the normal ranges. Ultrasonography and computed tomography of the abdomen showed a large mass of 40 × 25 cm, which extended from the posterior wall of the stomach to the spleen, enveloping the body and the tail of the pancreas (Figures 1 and 2). Moreover, the mass was in close proximity to the left colic flexure.

After appropriate fluid resuscitation and positioning of a naso-gastric tube, the patient was urgently taken to the operating room for exploration. A bilateral subcostal approach was chosen. The giant mass occupied the central abdomen and displaced the stomach anteriorly. The tumor abutted surrounding structures closely, especially the left colic flexure, which was compressed. There was no evidence of liver or peritoneal metastasis. After division of the gastro-colic ligament, it became clear that the giant mass derived from the posterior wall of the stomach and encased the body and the tail of the pancreas without infiltrating the celiac trunk or the mesenteric vessels. An intraoperative biopsy of the mass revealed an uncertain ‘spindle cell tumor’.

**Figure 3:**
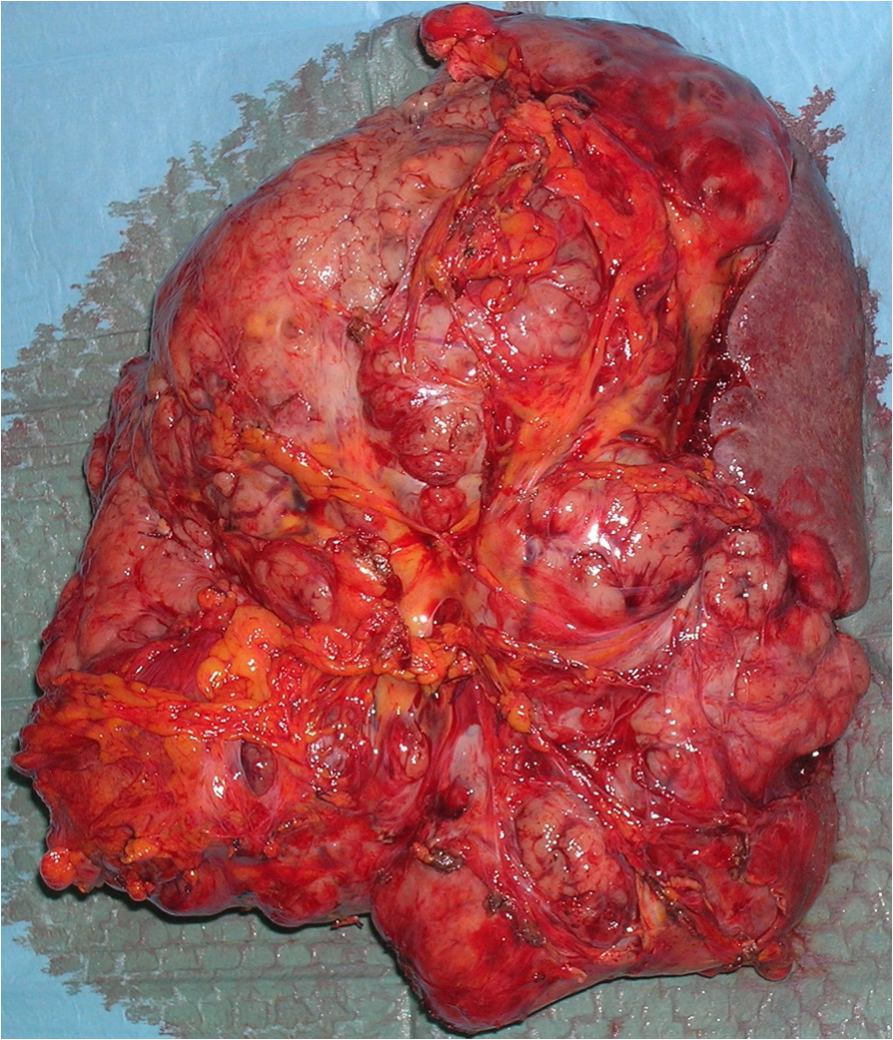
The removed tumor.

Once the technical feasibility of the intervention had been established, the splenic vessels were ligated at the superior margin of the pancreas, and the patient was subjected to en-block resection of the mass by distal spleno-pancreatectomy and sleeve resection along the large curve of the stomach. The mass was solid, measured 37 × 24 × 13 cm, weighed 8.5 kg, and had cyst and hemorrhagic areas; it did not infiltrate the spleen or pancreas (Figure 3).

Final histopathology of the resected specimen showed a spindle cell cancer with a mitotic count of 5/50 high-power field and a high proliferation index Ki67/Mib1 >10%. Immunohistochemistry revealed that the tumor was positive for CD 117/c-kit and for CD 34, and negative for desmin, vimentin, smooth muscle actin, muscle-specific actin, S-100 protein, and neuron-specific enolase. Hence, the final diagnosis was giant GIST of the stomach with high risk of malignancy.

The postoperative course was uneventful and treatment with imatinib mesylate 400 mg once daily started immediately. The patient appears to be disease free after four years.

## Discussion

Gastrointestinal stromal tumors (GISTs) are rare mesenchymal neoplasms with uncertain biological behavior that arise in the wall of the gastrointestinal tract. Despite recent progress made in the diagnosis of these tumors, their nosological classification remains challenging, owing to their high phenotypic polymorphism, and their ability to acquire a wide range of clinical phenotypes, from indolent-benign to malignant with high metastatic capacity. This peculiarity reflects the inability to predict the outcome of patients with these tumors and strongly limits prognostic evaluation.

GISTs may present in a number of different ways and are often diagnosed incidentally. Symptoms caused by GISTs are related to their location, leading to both mass effects and intraluminal bleeding. Large GISTs may cause vague abdominal discomfort, pain, bloating, early satiety, and, in rare cases, occlusion, compression of surrounding structures, and peritonitis due to spontaneous rupture into the peritoneum [8,9]. In some cases, the core of a large tumor leads to an intralesional degeneration, necrosis, or abscess development [10]. We report the case of a very large GIST of the stomach with an uncommon presentation, bowel obstruction due to left colic flexure compression.

GISTs, together with other soft tissue tumors, should be considered in the differential diagnosis of patients with an uncertain abdominal mass, even if a pathological diagnosis of GIST is not certain before or during surgery. For an uncertain abdominal mass, preoperative biopsy is commonly performed, but for GISTs the consideration for biopsy should be based on the extent of the disease. For localized or potentially resectable GISTs, for which preoperative imatinib is not being considered, preoperative pathological confirmation should not be necessary [1]. A biopsy is advisable for definitively unresectable or metastatic disease and in cases where preoperative therapy with imatinib is indicated because of marginally resectable disease or potentially resectable disease in high-risk patients [1]. Endoscopic ultrasound-guided fine-needle aspiration biopsy is preferred over percutaneous biopsy, owing to limited hemorrhage, the risk of tumor rupture, and dissemination. However, many pathologists might not produce a diagnosis using a fine-needle aspirate and a core-needle biopsy might be inconclusive if a necrotic or hemorrhagic portion of the tumor is sampled [1].

In our case, in spite of the large size, we performed only an intraoperative biopsy of the mass, as we needed to proceed with surgical excision, owing to the concomitant bowel obstruction. The intraoperative pathology was uncertain as it only showed ‘spindle cell cancer’.

After removal of any suspected GIST, postoperative pathology assessment is essential to confirm the diagnosis and to determine the correct size and the mitotic index, which are very important for the stratification of risk [11].

Surgery remains the therapy of choice for patients with primary GIST with no evidence of metastasis, and should be the initial therapy if the tumor is technically resectable and associated with an acceptable morbidity risk [1]. The goal of the operation is complete gross resection with a negative microscopic margin (R0 resection) without bleeding and rupture of the pseudocapsule [12].

The detection of microscopically positive margins in the case of large GISTs (>10 cm) is of dubious value, since during surgical maneuvers there is a possible exfoliation of neoplastic cells directly into the peritoneum [13]. Furthermore, there is no clear association between microscopically positive margins and poorer survival outcomes. Excision should be considered on a case-by-case basis, and often depends on the extremity of the first operation, as judged by the multidisciplinary team.

The decision to use preoperative imatinib is justified for locally advanced GISTs and for unresectable recurrent or metastatic disease [1]. Preoperative imatinib is also an option to facilitate function-preserving surgery for tumors in the gastro-esophageal junction and rectum [14,15]. The role of preoperative imatinib for treating primary localized GIST is questionable, as there is no clear evidence on the role of preoperative imatinib to obtain R0 resection. It is believed that exposure to preoperative imatinib may lead to a downregulation of c-kit expression with consequently selection of imatinib resistant clones of cancer cells, which may preclude the benefit of the surgical resection and the adjuvant therapy [16]. The role of new drugs (sunitinib) is still under evaluation in patients with progressive disease [17]. However, the optimal duration of preoperative therapy and the optimal timing of resection remain unknown. Another dangerous side effect can be the intralesional hemorrhage inside a giant mass, with hemoperitoneum or perforation [18].

## Conclusions

The surgical management of a giant GIST is a complex issue. We recommend that patients be continually evaluated by the surgical team for a possible resectability because we believe that the best strategy is ‘surgery when possible’, aimed at obtaining an R0 resection when possible. Even in this case of giant GIST of the stomach, an R0 resection can be safely achieved.

## Consent

Written informed consent was obtained from the patient for publication of this case report and accompanying images. A copy of the written consent is available for review by the editor-in-chief of this journal.

## Abbreviations

CA 19–9: Carbohydrate antigen 19–9; CD 34: Cluster of differentiation 34; CD 117/c-kit: Cluster of differentiation 117/proto-oncogene c-kit; CEA: Carcinoembryonic antigen; GI: Gastrointestinal; GISTs: Gastrointestinal stromal tumors; KIT: Tyrosine-protein kinase kit; PDGFRa: Platelet-derived growth factor receptor a.

## Competing interests

The authors declare that they have no competing interests.

## Authors’ contributions

AC and GP drafted the article; FC, AC, ELM, VC supervised the writing of the paper. All authors read and approved the final manuscript.
